# Targeting Neuroinflammation After Traumatic Brain Injury: Addressing Sex Differences to Improve Clinical Translation

**DOI:** 10.3390/ph19101659

**Published:** 2026-10-20

**Authors:** Caitlin Anne Sharratt, Jessica Leigh Hall, Peter John Crack, Hui Xin Tan

**Affiliations:** Department of Biochemistry and Pharmacology, Bio21 Molecular Science and Biotechnology Institute, University of Melbourne, Parkville, VIC 3010, Australia; caitlin.sharratt@student.unimelb.edu.au (C.A.S.); jessica.hall@student.unimelb.edu.au (J.L.H.); pcrack@unimelb.edu.au (P.J.C.)

**Keywords:** traumatic brain injury, neuroinflammation, sex-dependent differences, sex

## Abstract

Traumatic brain injury (TBI) is a leading cause of death and disability worldwide. Following the initial mechanical insult, a complex secondary injury cascade is triggered, resulting in neuroinflammation that can exacerbate neuronal damage and contribute to long-term neurological and psychiatric complications. Despite the development of numerous promising therapies targeting neuroinflammation after TBI, none have successfully translated from preclinical research into clinical practice. This persistent translational gap reflects limitations in preclinical and clinical study design, including inadequate representation of the biological heterogeneity of clinical TBI populations. This review critically evaluates the current landscape of therapies modulating neuroinflammatory pathways following TBI, with particular focus on how biological sex can influence therapeutic efficacy. Across multiple preclinical studies, sex-dependent differences were identified in molecular, histological, and functional responses to therapies targeting central regulators of neuroinflammation, indicating that biological sex can influence therapeutic efficacy across multiple inflammatory pathways. The influence of biological sex may also extend beyond preclinical development into clinical trials, where the underrepresentation of females and the failure to incorporate preclinical evidence of sex-dependent pharmacokinetics and therapeutic responses may represent potential contributors to variable or unsuccessful outcomes. Collectively, the evidence reviewed here suggests that biological sex may represent an important, yet underappreciated, contributor to the challenges of therapeutic translation and may obscure clinically meaningful treatment effects in defined patient subgroups. The findings presented throughout this review therefore suggest that biological sex should be considered a core component of therapeutic development, rather than solely as a secondary exploratory variable, to better characterise sex-dependent treatment responses and aid the clinical translation of therapies targeting neuroinflammation after TBI.

## 1. Introduction

Traumatic brain injury (TBI) represents a major global health challenge and remains one of the leading causes of death and disability in young and middle-aged adults [1]. Beyond acute mortality, the long-term functional burden of TBI is considerable. In 2021 alone, moderate to severe TBIs accounted for over 29 million years lived with disability worldwide, reflecting the persistent cognitive, behavioural, and physical impairments that commonly follow TBI [2]. Furthermore, extensive epidemiological evidence identifies TBI as an independent risk factor for several neurodegenerative and psychiatric disorders, including dementia, Alzheimer’s disease and depression [3,4,5,6,7]. Clinical studies have also consistently demonstrated sex differences in TBI outcomes, with females frequently experiencing poorer quality of life, greater psychiatric morbidity, and more persistent post-concussive symptoms than males [8,9,10].

These long-term neurological sequelae are largely driven by secondary injury processes initiated after the primary mechanical insult, which culminate in neuroinflammation that, when unresolved, can persist in a maladaptive self-sustaining cycle. Unlike the acute neuroinflammatory response, which is essential for tissue repair, chronic neuroinflammation promotes progressive white matter degeneration and neuronal dysfunction, contributing to ongoing neurological decline long after the initial injury [11,12,13]. Importantly, growing experimental evidence demonstrates that neuroinflammatory responses differ between males and females, with sex-dependent differences reported in microglial activation and inflammatory signalling pathways [14,15,16,17].

Despite considerable preclinical success, no pharmacological therapy targeting secondary neuroinflammatory mechanisms has translated into an approved treatment for TBI, and current clinical management remains limited to acute stabilisation and supportive care [18]. Numerous factors contribute to this persistent translation gap, many of which have been comprehensively reviewed elsewhere. These include inadequate consideration of the clinical heterogeneity of TBI populations, such as patient age, injury type and injury severity, as well as limitations of animal models and experimentally defined therapeutic windows that may not reflect realistic clinical timeframes [19,20,21]. However, comparatively little attention has been given to biological sex as a potential factor influencing therapeutic translation. The historical reliance on male-only animal models and the inadequate consideration of sex-dependent differences throughout the therapeutic development pipeline may represent an important, yet underappreciated, barrier to successful translation. As a result, clinically meaningful sex-dependent treatment responses may remain undetected until late-stage clinical testing, where they could contribute to inconsistent or unsuccessful outcomes.

The purpose of this review is to summarise the current landscape of pharmacological therapies targeting neuroinflammatory mechanisms following TBI and critically evaluate evidence for sex-specific therapeutic responses across recent preclinical studies and clinical trials. This review examines sex as one potentially important and modifiable factor within the wider challenges of TBI therapeutic development. We therefore assess how biological sex has been incorporated across the therapeutic pipeline and whether sex-dependent differences in treatment efficacy, pharmacokinetics, and underlying neuroinflammatory mechanisms may have implications for clinical translation.

We argue that incorporating sex as a fundamental biological variable throughout preclinical and clinical therapeutic development may aid in the translation of therapies targeting neuroinflammation after TBI.

## 2. Neuroinflammation in Traumatic Brain Injury

TBI pathophysiology is classically described as a biphasic process: an immediate primary insult followed by a delayed secondary cascade. The primary injury results directly from a mechanical force at the moment of impact and is largely irreversible, causing structural damage including contusions, diffuse axonal injury, and intracranial haemorrhage [22,23].

In contrast, the secondary injury phase develops over hours to months after the initial trauma and involves a series of complex interconnected inflammatory biochemical cascades resulting in neuroinflammation. A central feature of secondary injury is the disruption of metabolic homeostasis and ionic regulation. Mechanical deformation of neurons triggers the excessive release of glutamate, resulting in the overactivation of N-methyl-D-aspartate receptor (NMDA) and α-amino-3-hydroxy-5-methyl-4-isoxazolepropionic acid (AMPA) receptors, driving a pathological influx of calcium and sodium ions into neurons [24]. The resulting ionic imbalance drives ATP depletion as neurons attempt to restore ionic homeostasis, leading to metabolic failure, mitochondrial dysfunction, and excessive reactive oxygen species (ROS) production [25,26]. Together, these processes compromise blood-brain barrier (BBB) integrity, facilitate infiltration of peripheral immune cells and promote cerebral oedema [26].

Metabolic crisis and BBB breakdown collectively trigger a rapid neuroinflammatory response as damaged neurons, glia, and vascular cells release damage-associated molecular patterns (DAMPs), activating innate immune signalling pathways [27]. Microglia, the resident immune cells of the central nervous system (CNS), are among the first responders, undergoing morphological and transcriptional activation mediated by transcription factors, including interferon regulatory factors (IRFs), signal transducers and activators of transcription (STATs), and nuclear factor kappa-light-chain-enhancer of activated B cells (NF-κB) [28,29]. Activated microglia subsequently release a milieu of pro-inflammatory mediators, including tumour necrosis factor α (TNF-α), interleukin-6 and -1β (IL-6 and IL-1β), amplifying inflammation and promoting the recruitment and activation of peripheral immune populations, including monocyte-derived macrophages and lymphocytes [30].

The secondary injury cascade is normally a tightly regulated physiological response that is essential for tissue repair following CNS injury [27]. During the acute phase, neuroinflammation facilitates immune cell recruitment, clearance of cellular debris, release of neurotrophic factors, and activation of repair mechanisms that promote neuronal survival and recovery [11,31]. In response to the initial pro-inflammatory response, microglia can exert neuroprotective effects by secreting anti-inflammatory cytokines, including IL-4, IL-10 and IL-13, as well as producing proteins such as insulin-like growth factor 1 (IGF-1) and vascular endothelial growth factor (VEGF) to promote tissue repair and regeneration [29,32,33].

However, in some individuals, the secondary injury cascade fails to resolve, resulting in chronic neuroinflammation that can persist for years and drive progressive tissue damage, white matter degeneration, and neuronal loss [34]. Chronic neuroinflammation has therefore been strongly implicated in the development of the neurodegenerative and psychiatric disorders that occur after TBI [4,5,13,35]. Indeed, nationwide studies in Denmark and Finland found that a history of TBI increased the risk of dementia by up to 1.9-fold, and the risk of developing Alzheimer’s disease by 16%, while a meta-analysis of 57 clinical studies reported that prior TBI was associated with a 1.55-fold increased risk of neurological disorder and a twofold increased risk of psychiatric disorder [4,5,7].

Although the mechanisms underlying the transition from acute to chronic neuroinflammation remain incompletely understood, persistent microglial activation is considered a key driver of this process [27]. In some experimental TBI models, activated microglia persist for at least one year after injury, coinciding with progressive lesion expansion, hippocampal neurodegeneration, demyelination, and impaired spatial learning and memory [36,37,38]. Similarly, analysis of the human Glasgow TBI Archive identified densely packed, reactive microglia in 28% of individuals who survived up to 18 years after a single moderate or severe TBI, accompanied by ongoing white matter degeneration [13]. This sustained glial activation is even more pronounced after multiple traumatic brain injuries [39,40]. It is of significant note then that accumulating evidence demonstrates that microglial biology has fundamental sex differences at the molecular, morphological, transcriptional, and functional levels, with microglia exhibiting distinct roles in brain development, sexual differentiation, and inflammatory responses [14,15,41,42,43]. Critically, these biological differences at a cellular level are mirrored by clinically meaningful differences in recovery following TBI between males and females. It is therefore likely that biological sex is a fundamental determinant of the neuroinflammatory phenotype post-TBI, and consideration of sex as a biological variable throughout therapeutic development is critical.

### Sex Differences in Human TBI Recovery

Biological sex influences numerous aspects of brain development and function, as well as both innate and adaptive immunity [44,45]. These differences arise through complex interactions between sex chromosome genes, sex hormones, epigenetic regulation and environmental factors, producing sex-dependent inflammatory and neurobiological responses throughout life [45,46]. Despite this, both preclinical and clinical research into sex differences following TBI has largely focused on the effects of the ovarian hormones, namely oestrogen and progesterone. As these hormones decline markedly following menopause, many clinical studies have compared outcomes between pre- and post-menopausal females to investigate whether ovarian hormones influence recovery after TBI, while other biological determinants of sex differences remain comparatively understudied [47,48].

This ovarian hormone-centred approach has provided a framework for identifying some biological differences between males and females but does not completely explain them, which may contribute to discrepancies in injury outcomes reported in the clinic. Some studies found no differences between males and premenopausal females, with improved outcomes observed only in postmenopausal females, including lower mortality and better cognitive performance [49,50,51]. Conversely, other studies have reported lower mortality in premenopausal females compared with males [52,53,54]. However, these findings are often attributed to the presumed neuroprotective effects of oestrogen and progesterone without validating their causal relationship within the same patient population [55,56]. Instead, these interpretations are largely based on preclinical studies, potentially overlooking the wider biological mechanisms underlying sex-dependent differences following TBI. Notably, when outcomes beyond mortality are considered, including functional recovery, quality of life, persistent post-concussive symptoms and the development of neuropsychiatric disorders, a growing body of evidence consistently demonstrates that females experience poorer long-term outcomes following TBI than males [8,9,10,57,58,59].

This evidence spans all injury severities. In mild TBI, the multinational Collaborative European NeuroTrauma Effectiveness Research in TBI study (*n* = 2864) reported significantly worse 6-month functional outcomes on the Glasgow Outcome Scale, lower health-related quality of life, and poorer mental health outcomes [8]. These differences were not explained by gender-associated sociodemographic factors or differences in healthcare pathways. Similarly, the North American Prospective Transforming Research and Clinical Knowledge in Traumatic Brain Injury study (*n* = 2000) found that females experienced greater post-concussive symptom severity 12 months after injury [10]. Additionally, a prospective cohort from the University of Rochester (*n* = 1425) reported significantly greater odds of persistent post-concussive symptoms in females 3 months after mild TBI [57].

Sex differences in recovery are also evident following moderate and severe TBI. This is supported by a prospective cohort study from a Los Angeles trauma centre (*n* = 313), which found that females were 1.57 times more likely to experience poor functional outcomes 18 months after injury than males [58]. Likewise, a German cross-sectional study (*n* = 135) reported that females over 50 years of age had significantly lower quality of life and greater symptoms of depression, anxiety, and post-traumatic stress for up to 10 years following injury compared with age-matched males [59]. Collectively, these findings are supported by a meta-analysis of 8 clinical studies, which found that females experienced poorer outcomes in 85% of assessed variables across all TBI severities, including post-concussive symptoms, affective disturbances, post-traumatic amnesia, and duration of hospitalisation [9].

Overall, current clinical evidence identifies biological sex as an independent determinant of functional, neuropsychiatric and quality-of-life outcomes following TBI. Despite this, sex has rarely been incorporated into the design, analysis, or interpretation of preclinical and clinical trials evaluating emerging therapies targeting neuroinflammation. As a result, clinically relevant sex-specific differences in therapeutic efficacy may have gone undetected, contributing to challenges in translating promising preclinical therapies into effective clinical treatments.

## 3. Proteins Involved in Neuroinflammation Targeted in Preclinical Studies

**Figure 1:**
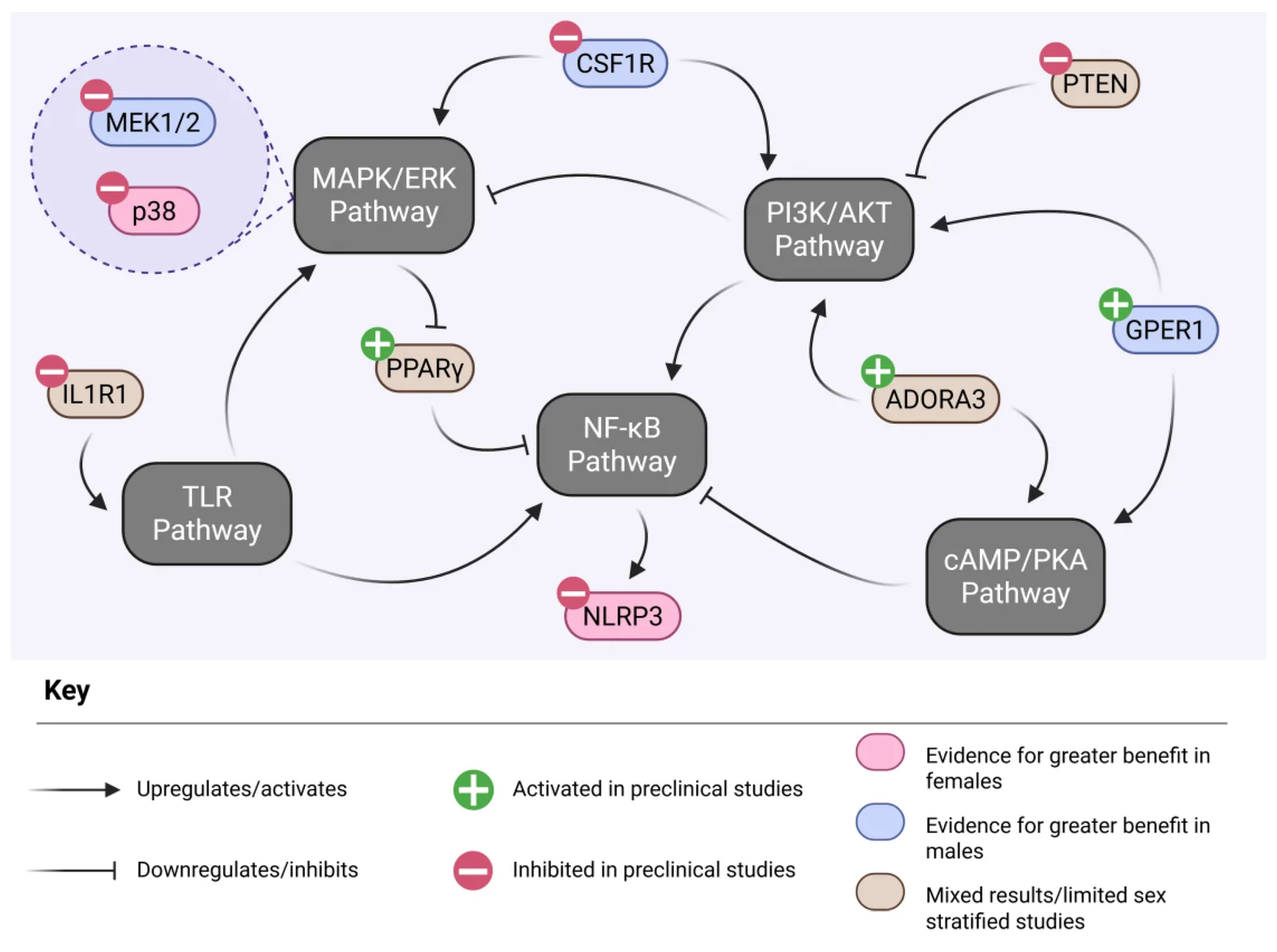
Overview of targeted neuroinflammatory signalling pathways, their interactions following traumatic brain injury, and sex-dependent differences in therapeutic efficacy. Neuroinflammation is regulated by a highly interconnected network of signalling pathways, including TLR, MAPK/ERK, NF-κB, PI3K/Akt and cAMP/PKA. The TLR, MAPK/ERK and NF-κB pathways regulate innate immune activation, pro-inflammatory cytokine production, oxidative stress, apoptosis, and blood-brain barrier disruption. The PI3K/Akt pathway facilitates neuronal and microglial survival and tissue repair, while the cAMP/PKA pathway promotes transcription of anti-inflammatory genes and neuronal survival. Although these pathways are conserved between sexes, recent preclinical studies indicate that their activation, regulation and downstream effects may be sex-dependent, contributing to differences in neuroinflammatory responses following traumatic brain injury. Individual proteins were coloured to indicate a trend towards greater therapeutic benefit observed in one sex (pink: female, blue: male and brown: mixed results) reported by one or more preclinical studies that included females within their study. They should not be interpreted as statistically conclusive evidence of sex-dependent therapeutic efficacy. Created in BioRender. Wong, B. (2026) https://BioRender.com/zw1m5zd (accessed on 13 August 2026).

Numerous pathways involved in the regulation of the neuroinflammatory response have been identified as potential therapeutic targets following TBI (Figure 1). While proteins within pathways are discussed individually throughout this review, they should not be viewed as isolated mediators. Neuroinflammation is regulated by a highly interconnected network of pro- and anti-inflammatory signalling pathways, in which modulation of one pathway can influence multiple downstream and parallel mechanisms. Consequently, targeting a single protein frequently affects several aspects of secondary injury, including immune cell activation, cytokine production, oxidative stress, BBB integrity, and apoptosis [60]. Likewise, sex-specific differences within one neuroinflammatory pathway are unlikely to occur in isolation but instead propagate throughout the broader inflammatory network. Understanding these interactions is therefore essential when interpreting the therapeutic potential of individual protein targets.

To summarise the current landscape of therapies targeting neuroinflammation after TBI, a narrative literature review was conducted on recent preclinical studies to identify studies investigating the pharmacological or genetic modulation of specific target proteins following experimental TBI in rodents. Studies published since 2020 that evaluated therapeutic modulation and reported functional, histological, or molecular outcomes were considered. Studies were excluded where there was insufficient evidence linking the target protein to neuroinflammatory signalling or insufficient reproducible preclinical evidence supporting its therapeutic potential. All eligible target proteins were considered, and targets were included in the review if at least one preclinical study included female rodents. Targets for which no preclinical studies had evaluated females were therefore excluded, as the absence of female data prevented assessment of potential sex-dependent therapeutic responses. This approach was used to minimise selection bias towards targets with established sex differences and instead evaluate the extent to which sex-dependent therapeutic responses are evident across the broader landscape of neuroinflammatory targets. The selected studies are summarised in Table 1 and their broader interactions within the neuroinflammatory response to TBI are illustrated in Figure 1.

As much of the existing research predominantly focuses on the effects of ovarian hormones, particularly oestrogen and progesterone, on causing sex-dependent responses to therapies, the current literature does not comprehensively capture the biological mechanisms underlying sex-dependent therapeutic responses. The lack of understanding of how the interaction of sex chromosome genes and epigenetic regulation can influence neuroinflammation following TBI represents an important limitation when interpreting sex-dependent responses across the selected therapeutic targets.

### 3.1. MEK1/ERK Pathway

Mitogen-activated protein kinase kinase 1 and 2 (MEK1/2) are activated downstream of multiple cell-surface receptors, including glutamate-activated NMDA receptors, and regulate extracellular signal-regulated kinase (ERK)-dependent transcription of pro-inflammatory cytokines and pro-apoptotic signalling proteins [81,82]. Given their central role in coordinating inflammatory responses, pharmacological inhibition of MEK1/2 has been investigated using compounds including U0126 and trametinib. Collectively, these studies demonstrated reduced expression of pro-inflammatory cytokines, including IL-1β, TNF-α, and IL-12, together with improvements in learning, memory, and motor function following experimental TBI [62,63,83]. Importantly, a study that included both sexes demonstrated comparable therapeutic benefits in both females and males but appeared to confer greater behavioural recovery in males. However, this difference was not statistically tested between sexes [62].

### 3.2. p38 MAPK

In addition to MEK1/2, p38 mitogen-activated protein kinase (MAPK) is another key regulator of the ERK pro-inflammatory pathway [84]. Activated downstream of MAP kinase kinase 3/6 (MKK3/6) in response to DAMPs, pro-inflammatory cytokines and glutamate release, p38 MAPK promotes the expression of inflammatory mediators including IL-1β, TNF-α, and IL-6, as well as enzymes involved in oxidative stress, tissue remodelling, and leukocyte recruitment [85].

Pharmacological inhibition of p38 MAPK has been investigated as a strategy to attenuate secondary neuroinflammation. Acute administration of a small-molecule p38 MAPK inhibitor preserved synaptic integrity and improved motor coordination, anxiety-like behaviour, and depression-like behaviour following experimental TBI [61]. In contrast to the greater therapeutic responses observed in males following MEK1/2 inhibition, p38 MAPK inhibition appeared to confer greater long-term benefit in females. However, this apparent sex difference was not statistically tested. Female mice exhibited reduced synapse loss, decreased pro-inflammatory microglial activation, and significant improvements in motor function and anxiety-like behaviour up to 4 weeks after injury, whereas male mice showed only partial recovery of motor deficits with no significant improvements in anxiety-like behaviour or synaptic preservation [61].

This is supported by evidence from experimental models of CNS autoimmunity which found that genetic ablation of p38 MAPK protected females but not males and, in some models, promoted a more pro-inflammatory phenotype in male animals [86,87]. Furthermore, deletion of an oestrogen receptor in p38-deficient myeloid cells abolished protection in females while promoting protection in males, demonstrating that interactions between p38 MAPK signalling and sex hormone pathways can fundamentally alter therapeutic responses [88].

### 3.3. GPER

Beyond inhibiting pro-inflammatory signalling, therapeutic strategies have also sought to activate anti-inflammatory pathways. G protein-coupled oestrogen receptor (GPER) is a membrane-bound receptor activated by the endogenous ligand 17β-oestradiol and the selective agonist G-1 [89]. Activation of GPER promotes anti-inflammatory and neuroprotective signalling by stimulating adenylyl cyclase, increasing intracellular cyclic adenosine monophosphate (cAMP) and subsequent protein kinase A (PKA) activation and promoting cyclic AMP response element-binding protein (CREB)-mediated transcription of anti-inflammatory and cell survival genes [90]. In addition, GPER modulates the MAPK/ERK, Phosphoinositide 3-kinase and Protein Kinase B (PI3K/Akt) pathways via epidermal growth factor receptor transactivation [91].

Pharmacological activation of GPER has been investigated as a strategy to limit neuroinflammation following TBI. Across preclinical studies, GPER agonism reduced cerebral oedema, BBB permeability, and pro-inflammatory cytokine production while increasing anti-inflammatory cytokine expression during the acute phase of injury [69,70]. These molecular changes were accompanied by improvements in anxiety-like behaviour and memory in both male and ovariectomised female mice, in which surgical removal of the ovaries markedly reduced circulating oestrogen and progesterone levels, indicating that GPER-mediated neuroprotection is influenced by the ovarian hormonal environment. In contrast, intact female mice exhibited increased pro-inflammatory cytokine expression and reduced anti-inflammatory cytokine production following GPER agonism [70]. In addition, it was shown that ovariectomised females had significantly higher baseline GPER expression than intact females. Furthermore, oestradiol supplementation abolished the beneficial effects of GPER activation in ovariectomised females, suggesting that circulating oestrogen levels directly influence GPER-mediated inflammatory signalling. Mechanistically, the protective effects of GPER agonism in males and ovariectomised females were mediated through PI3K/Akt signalling, as pharmacological inhibition of Akt abolished these neuroprotective responses [70].

### 3.4. CSF1R

Instead of directly modulating inflammatory signalling, another therapeutic strategy aims to limit microglial proliferation. Colony Stimulating Factor 1 Receptor (CSF1R) regulates microglial survival, proliferation and differentiation through activation of PI3K/Akt, MAPK/ERK and NF-κB signalling pathways [92]. Pharmacological inhibition of CSF1R has therefore been investigated as a means of transiently depleting activated microglia and promoting microglial turnover following TBI. CSF1R inhibition improved neuronal survival, reduced lesion volume, and enhanced functional outcomes following both acute and chronic treatment [73,74,75,76]. However, therapeutic responses to CSF1R inhibition differed between sexes. Although pharmacological inhibition similarly reduced microglial numbers and inflammatory marker expression in both male and female mice, increased neuronal cell numbers and improved neurological severity scores were observed only in males [73]. In the chronic stages of injury, CSF1R inhibition reduced lesion size in both males and females. However, in the acute and sub-acute stages of injury, CSF1R inhibition alone had no effect, and males only showed benefits when CSF1R inhibition was combined with IL1R1 inhibition [73,76]. Furthermore, CSF1R inhibition worsened motor coordination in females but had no effect in males [73]. At the molecular level, CSF1R inhibition increased expression of the pro-inflammatory genes *Bax* and *Il1r1* in females, whereas both were reduced in males. Interestingly, females also exhibited increased expression of the traditionally anti-inflammatory genes *Mrc1* and *Arg1*, while expression of these genes either decreased or remained unchanged in males [73]. Similar male-specific benefits of CSF1R-mediated microglial depletion have been reported in rodent models of sleep deprivation, neuropathic pain, and single prolonged stress [93,94,95].

Wang et al. proposed that the sex-dependent effects of CSF1R and IL1R1 inhibition following TBI may be related to peripheral myeloid infiltration [76]. Peripheral myeloid infiltration into the brain following TBI has been shown to be greater in males than in females [96]. As IL1R1 contributes to the trans-endothelial migration of leukocytes and CSF1R regulates microglial proliferation, Wang et al. hypothesised that the interaction between resident microglia and infiltrating myeloid cells may contribute to the greater therapeutic response observed in males.

### 3.5. NLRP3 Inflammasome

Another major therapeutic target for limiting microglial inflammatory activity is the nucleotide-binding oligomerisation domain-like receptor protein 3 (NLRP3) inflammasome. NLRP3 is an intracellular pattern recognition receptor predominantly expressed by microglia that is activated in response to DAMPs and transcriptionally primed by NF-κB signalling [97]. Upon activation, NLRP3 assembles the inflammasome complex, leading to caspase-1 activation, release of IL-1β and IL-18, and induction of pyroptotic cell death. Across multiple preclinical studies, pharmacological inhibition or glia-specific deletion of NLRP3 consistently reduced IL-1β and IL-18 expression, brain water content, and lesion volume while improving neuronal survival during the acute phase of injury [66,67,68]. These anti-inflammatory effects were accompanied by improved memory and reduced depression-like behaviours at chronic time points, demonstrating sustained benefits beyond the acute inflammatory response. Importantly, the limited studies that evaluated both sexes suggest that modulation of NLRP3 also has sex-dependent effects. Astrocyte-specific deletion of NLRP3 improved social interaction and reduced depression-like behaviours in both male and female mice, although reductions in astrocyte and microglial populations were significantly greater in females than in males [66].

### 3.6. Implications of Sex Differences for Therapeutic Development

The studies reviewed here indicate that biological sex influences therapeutic responses across multiple central regulators of the neuroinflammatory response following TBI. Sex-dependent differences were observed not only in behavioural outcomes but also at the molecular and histological levels, indicating that males and females might engage distinct inflammatory mechanisms when targeting the same protein. As these proteins occupy key regulatory positions within highly interconnected neuroinflammatory signalling networks, sex-specific modulation of one pathway is likely to influence numerous downstream inflammatory processes and therapeutic targets. Biological sex should therefore be considered a fundamental determinant of neuroinflammation rather than an isolated variable affecting individual therapeutic targets.

Currently, the overwhelming majority of preclinical TBI studies continue to evaluate therapies exclusively in male animals, with an estimated 86–92% excluding female animals [51,98,99,100]. This bias originated from the assumption that fluctuations in ovarian hormones introduced excessive experimental variability in female animals. However, this has since been comprehensively refuted, with multiple studies demonstrating that female rodents are no more variable than males across a broad range of biomedical and neurological research [101,102,103]. The continued underrepresentation of females therefore represents a persistent limitation in preclinical TBI research rather than an evidence-based experimental strategy.

Failure to account for biological sex during preclinical development has implications beyond determining whether a therapy is effective in both sexes. Many neuroinflammatory pathways are directly or indirectly regulated by sex hormones and sex-related genes and epigenetic regulation, resulting in sex-dependent inflammatory signalling, microglial activity and pharmacological responses [104,105]. As such, the molecular mechanisms underlying therapeutic benefit, as well as optimal dosing regimens, therapeutic windows, and treatment efficacy, may differ between males and females. Without rigorously investigating these differences before clinical testing, promising therapies may advance using dosing strategies that are suboptimal for one sex, while genuinely effective treatments may be passed over when male-only analyses obscure clinically meaningful sex-specific responses. Addressing this knowledge gap may aid in the successful translation of neuroinflammatory therapies for TBI.

## 4. The Translation Gap

The failure of promising therapies targeting neuroinflammation in clinical trials is frequently interpreted as evidence that the targeted pathway lacks therapeutic value, prompting the search for new targets. However, the evidence discussed throughout this review suggests that many translational failures may instead reflect limitations of the preclinical therapeutic development pipeline rather than shortcomings of the therapeutic targets themselves. One important limitation of current preclinical research is the limited consideration of biological sex. Despite evidence that sex may influence therapeutic responses across multiple central regulators of neuroinflammation, many preclinical studies continue to assess therapies exclusively in male animals. As a result, many therapies progress towards clinical evaluation without a comprehensive understanding of how efficacy is influenced by biological sex, despite growing evidence that secondary injury mechanisms and recovery following TBI are sex-dependent [106]. Similar limitations extend to other important biological variables, including age and injury severity, which have been comprehensively reviewed elsewhere [107,108,109].

These limitations are compounded during clinical translation. A recent systematic review of registered TBI clinical trials conducted between 2004 and 2023 reported that 22.1% of trials were discontinued prematurely, most commonly during Phase II because of inadequate recruitment, funding limitations, or failure to demonstrate efficacy [110]. Furthermore, 75.2% of registered TBI trials enrolled fewer than 100 participants, substantially limiting statistical power for subgroup analyses. As a result, even when females are included in clinical trials, participant numbers are often insufficient to determine whether treatment efficacy differs between sexes. Consequently, potentially meaningful sex-specific therapeutic effects may remain undetected, leading promising therapies to be dismissed despite the possibility that they may significantly benefit one sex even if they do not benefit the other. Importantly, this limitation is not merely theoretical but is evident throughout the TBI clinical trial literature summarised in Table 2, where females remain underrepresented, sex-stratified efficacy analyses are frequently omitted, and trial designs rarely incorporate preclinical evidence of sex-dependent therapeutic responses or pharmacokinetics.

### 4.1. Underrepresentation of Females in Clinical Trials

The Erythropoietin in Traumatic Brain Injury trial provides a clear example of the persistent underrepresentation of females and the limited consideration of biological sex in TBI clinical trial design. The trial was conducted in collaboration with the Australian and New Zealand Intensive Care Society Clinical Trials Group (ANZICS-CTG) and investigated the effect of erythropoietin (EPO), a hormone that stimulates red blood cell production. Although EPO demonstrated robust neuroprotective and anti-inflammatory effects in experimental TBI, the largest clinical trial investigating EPO failed to improve functional neurological outcomes despite secondary analyses suggesting reduced mortality in patients without mass lesions [111,118]. However, only 16% of participants allocated to the treatment arm were female, preventing meaningful sex-stratified analyses of outcomes. This is particularly important given that preclinical studies have demonstrated sex-dependent responses to EPO, with male rodents exhibiting more rapid improvements in memory, sensorimotor function and neurogenesis than females following TBI [119]. As a result, it remains unknown whether pooling outcomes statistically masked sex-specific therapeutic efficacy. Unfortunately, an analysis of ANZICS-CTG studies conducted between 2014 and 2023 found that females represented only 36.3% of all enrolled participants, highlighting that underrepresentation of females is a persistent challenge in major clinical trials [120]. This points to a critical gap in recruitment guidelines, which should establish minimum requirements for female representation to ensure that trials are adequately powered to evaluate sex-specific treatment effects.

### 4.2. Failure to Account for Sex-Dependent Pharmacokinetics in Clinical Trials

Underrepresentation of females is only one aspect of the broader challenge of incorporating biological sex into TBI clinical trial design. Even when therapies are inherently linked to sex biology, differences in pharmacokinetics and pharmacodynamics between males and females are rarely considered, despite extensive evidence that these factors influence drug efficacy and safety [121]. The Phase III SYNAPSE and ProTECT III trials investigating progesterone illustrate the consequences of this oversight [112,113]. Despite more than 300 preclinical studies demonstrating the neuroprotective effects of progesterone in both male and female rodents, neither SYNAPSE nor ProTECT III demonstrated improvements in functional outcome or mortality following TBI [112,113,122,123,124]. Although approximately 22% of participants receiving progesterone in SYNAPSE were female, treatment outcomes were not stratified by sex. Comparatively, ProTECT III reported no significant sex-specific treatment effects. A subsequent evaluation identified multiple inconsistencies between the clinical trial protocols and the supporting preclinical evidence, including inadequate optimisation of dose, treatment duration, therapeutic window, and route of administration, all of which have been shown experimentally to influence progesterone efficacy in a sex-dependent manner [122]. Thus, the failure of these trials may reflect limitations in trial design rather than a lack of therapeutic potential.

Similar shortcomings are evident in other clinical trials. For example, the Phase III trial of tirilazad mesylate failed to demonstrate improvements in functional outcome following TBI, but subsequent evaluation revealed that females at several North American and European sites were routinely underdosed because body weight was estimated rather than measured, resulting in lower drug exposure [114,125]. Furthermore, recognised sex differences in tirilazad pharmacokinetics, including drug absorption, distribution, metabolism, and clearance, were not incorporated into the original trial design [114]. Interestingly, post hoc analyses identified a significant benefit in male patients with traumatic subarachnoid haemorrhage, whereas females experienced slightly higher mortality, an almost fourfold increase in shock, and a twofold increase in hypoxia compared with controls [114]. Recognition of these sex-specific differences subsequently prompted clinical trials in aneurysmal subarachnoid haemorrhage with altered dosing to account for pharmacokinetic differences between males and females. In these studies, tirilazad significantly improved recovery and reduced mortality, particularly in males, while dose escalation in a subsequent female-only trial significantly reduced delayed cerebral ischaemia and clinical vasospasm, despite not improving mortality or functional outcomes [126,127]. Together, these findings demonstrate that therapeutic efficacy can be substantially influenced by sex-specific pharmacokinetics and highlight the importance of incorporating dosing optimisation into clinical trial design rather than applying a uniform strategy to both sexes.

### 4.3. Inadequate Consideration of Preclinical Research in Clinical Trials

Beyond differences in pharmacokinetics and dosing, clinical trial design has also frequently failed to incorporate preclinical evidence demonstrating that therapeutic mechanisms themselves may be sex-dependent. In a Phase II clinical trial, doxycycline reduced serum neuron-specific enolase concentrations and improved Glasgow Coma Scale scores during the acute phase following TBI. However, these early benefits did not translate into improved long-term neurological outcomes [115]. These findings were not analysed according to sex, nor had doxycycline been evaluated in female preclinical TBI models before clinical translation. This is particularly noteworthy given that the closely related tetracycline antibiotic minocycline exhibits pronounced sex-dependent effects across multiple CNS injury models, including stroke and repeated mild TBI, where therapeutic benefit is largely restricted to males [116,128,129]. Given the similar mechanisms of action of these compounds, the potential for comparable sex-dependent responses to doxycycline should have been established before progression to larger efficacy trials.

Comparable limitations are evident in the clinical development of Ronopterin, a nitric oxide synthase inhibitor that failed to meet its primary endpoint in a Phase III TBI trial [117]. Although post hoc analyses suggested a possible therapeutic benefit when treatment was initiated within 12 h of injury, sex-stratified analyses have yet to be reported. This omission is particularly significant given the well-established interaction between nitric oxide signalling and oestrogen-mediated neuroprotection in experimental models of cerebral ischaemia [130,131]. Furthermore, Ronopterin has not been evaluated in female preclinical TBI models, preventing assessment of whether treatment efficacy differs between sexes before clinical translation.

## 5. Recommendations

Numerous reviews have proposed strategies to improve the clinical translation of therapies for TBI, including more comprehensive characterisation of secondary injury mechanisms, improved understanding of drug pharmacokinetics, optimisation of dosing regimens and therapeutic windows, and evaluation across multiple injury models and age groups [19,20,21]. However, comparatively little attention has been given to biological sex as a potential factor influencing therapeutic efficacy, despite growing evidence that males and females differ in TBI pathophysiology, neuroinflammatory signalling, and treatment responses. The findings presented throughout this review suggest that biological sex needs to become a core component of therapeutic development rather than a secondary exploratory variable to aid in the clinical translation of therapies targeting neuroinflammation after TBI.

Although existing mandates from funding bodies and journals are important steps towards addressing sex bias, this top-down approach has not been sufficient to achieve consistent sex inclusion across the research pipeline [132]. Further strategies are therefore needed to translate these policies into routine research practice. While novel frameworks and comprehensive guidelines for sex-inclusive research have been developed and reviewed elsewhere [133,134,135], the following sections outline additional strategies to promote sex inclusivity across individual, institutional, and educational settings.

### 5.1. Strengthen Upstream Mandates

Given the long history of the exclusion of females from preclinical and clinical studies, achieving meaningful change cannot rely on researchers voluntarily adopting sex-inclusive practices [100,136]. Researchers have been shown to adopt an exceptionalist view that their specific research does not require both sexes, despite established policies promoting sex inclusion [137]. Additionally, pressure from funding and publishing bodies to produce more ‘complete’ mechanistic narratives may incentivise researchers to prioritise generating increasingly complex datasets over validating findings in both sexes [138]. Sex inclusion, analysis, and reporting must therefore be embedded and enforced throughout the research pipeline, beginning with funding and ethics approval and extending through to publication.

Public funding bodies have taken important steps towards this goal. The US National Institutes of Health have implemented policies to include females as part of the Sex as a Biological Variable policy [139]. Similarly, research funded through the Australian National Health and Medical Research Council and Medical Research Future Fund is required to address sex and gender in research proposals under the Statement on Sex, Gender, Variations of Sex Characteristics and Sexual Orientation in Health and Medical Research [140]. However, loopholes and limited enforcement currently allow studies to deviate from approved sex-inclusion requirements after funding is awarded [138,141]. Stronger monitoring of compliance throughout the funded research period is therefore necessary to encourage adherence to the research proposed and promote discovery of therapeutics for both sexes.

One approach is to incorporate sex-inclusion checks into ethics approval and annual review processes. The Sex Inclusive Research Framework provides a decision tree and associated traffic-light system to identify proposals that lack balanced sex inclusion or appropriate sex-specific analysis plans [135]. Integrating such frameworks into ethics approvals and reviews could ensure that studies have adequate sex-inclusive designs and analysis plans before approval and that these requirements are maintained throughout the study.

Similarly, journals should require explicit reporting of sex for animals, cell lines and organoids, including sufficient justification for any exclusions. Despite the introduction of the international Sex and Gender Equity in Research guidelines in 2016, limited improvements in the inclusion and reporting of sex in published research have been observed [132,142]. Collectively, this suggests that more rigorous policies and enforcement are required. Without enforceable requirements and consequences for non-compliance at both the initiation and publication stages, sex inclusion will remain a recommendation rather than a standard of rigorous therapeutic development.

### 5.2. Sex-Inclusive Experimental Designs

Effective implementation of sex-inclusion policies requires actionable changes to experimental designs. Preclinical studies should include both sexes from the earliest stages of therapeutic development and be adequately powered to detect sex-specific treatment effects where possible. This principle extends beyond in vivo studies, as many commonly used immortalised cell lines are male-derived [143]. Diversifying cell line sources for in vitro experiments can therefore reduce sex bias and improve experimental rigour. Therapeutic outcomes should also be reported separately for males and females, with statistical interaction analyses used to determine whether treatment efficacy differs by sex. Mechanistic studies should follow the same standards, particularly research investigating how biological sex influences neuroinflammatory signalling and contributes to sex-dependent therapeutic responses and differences in TBI outcomes.

These principles should also extend throughout clinical translation. Clinical trials should strive to recruit sufficient numbers of both male and female participants to enable adequately powered sex-stratified analyses. However, this presents a practical challenge as females account for approximately 26% of reported moderate-to-severe TBI cases and around 36% of mild TBI cases [144]. In this context, early clinical trials should aim to evaluate potential sex-dependent treatment responses, with deliberate recruitment strategies implemented to ensure sufficient female representation for meaningful analysis. Once robust clinical evidence demonstrates that biological sex has minimal influence on treatment efficacy, subsequent trials may prioritise recruitment according to other eligibility criteria. Regardless of participant composition, trial protocols should incorporate available preclinical evidence of sex-dependent differences in therapeutic efficacy, dosing requirements, therapeutic windows, and pharmacokinetics to ensure that therapies are appropriately optimised for all participants.

Careful interpretation of trial data is equally important, as pooling males and females without appropriate subgroup analyses can statistically mask clinically meaningful treatment effects, leading potentially effective therapies to be prematurely abandoned [145]. Integrating biological sex into all stages of therapeutic development can allow researchers to identify sex-dependent therapeutic responses, minimise translational waste, and prevent promising treatments from being lost through inappropriate study design or statistical analysis.

### 5.3. Education, Training and Cultural Change

Many researchers continue to hold misconceptions about the biological basis and relevance of sex differences, with sex often considered primarily in the context of ovarian hormones rather than sex chromosomes, epigenetic regulation, and their interactions [100,137]. Concerns that ovarian hormone fluctuations make females inherently more variable have also been repeatedly disproven, and routine tracking of the oestrous cycle is not necessary unless there is a specific biological rationale [101,102,103,146]. Addressing these misconceptions will require broader education and cultural change across the research community [136,138]. Institutional guidelines, workshops, and online training should therefore be provided and strongly encouraged to give researchers practical guidance on sex-inclusive experimental design, analysis, and interpretation.

For TBI research specifically, education should also emphasise that the objective of incorporating biological sex into TBI therapeutic research is not to develop therapies that produce identical responses in males and females but to understand how therapeutic efficacy differs between sexes and optimise treatment accordingly. Within this framework, a therapy that demonstrates efficacy predominantly in one sex should not be considered a translational failure. Instead, recognising and understanding sex-dependent therapeutic responses will enable more precise clinical application and improve the successful translation of therapies targeting neuroinflammation after TBI.

## 6. Conclusions

The persistent failure of promising TBI therapeutics in clinical trials highlights a fundamental need for change in how therapies are developed, evaluated, and translated. The studies reviewed here show that biological sex is a core determinant of neuroinflammatory signalling and therapeutic response, yet it continues to be overlooked throughout the therapeutic development pipeline. Improving the clinical translation of therapies targeting neuroinflammation will therefore require a shift away from a ‘one-size-fits-all’ approach towards recognising sex as a fundamental determinant of therapeutic efficacy. Achieving this shift will require a coordinated commitment from researchers, funding bodies, institutions, and journal editors to ensure that both sexes are systematically incorporated, analysed, and reported at every stage of therapeutic development, from preclinical target identification to clinical trial design and interpretation. Systematic incorporation of biological sex throughout preclinical and clinical development will help distinguish truly ineffective therapies from interventions whose efficacy, dose requirements or therapeutic windows differ between patient subgroups, thereby enabling a more precise approach to neuroinflammatory therapy after TBI.

## Figures and Tables

**Table 1:** Summary of preclinical studies in rodents targeting specific neuroinflammatory proteins following experimental traumatic brain injury.

Targeted Protein/s	Direction of Regulation	Sexes Tested	Glial Markers and Morphology	Neuronal Health	Brain Structure	Functional Outcomes	Other
P38 MAPK [61]	Inhibition (acute)	M & F	^^^↓ CD68 **(males only; females ↔)** ^^^*↓ TMEM119 (females only; males ↔) *↓ CD68 **(females only; males ↔)**	*↑ Synaptic connections **(females only; males ↔)**	-	*↑ Motor coordination (females only; males ↔) *↓ Depression-like behaviour *↓ Anxiety-like behaviour **(females only; males ↔)**	^^^↓ Eotaxin, GM-CSF, G-CSF, IL13, IL5, CCL4 (MIP1β), VEGF **(females only;** **males ↔)**
MEK 1/2 [62]	Inhibition (continuous)	M & F	-	-	-	^^^↑ Motor coordination **(males only; females ↔)** ^^#^↓ Neurological severity score ^#^↑ Motor coordination ^#^↑ Spatial memory	^#^↓ p-ERK, p-H3S10 (male effect larger than females, though not statistically tested)
MEK 1/2 [63]	Inhibition (continuous)	Not stated	^^^↑ Iba1+ cells ^^^↓ CD86+ cells ^#^↔ Iba1+ cells	^^^↔ Myelin integrity ^#^↑ Myelin integrity	-	^^^↔ Motor coordination ^^^↔ Spatial memory ^#^↑ Motor coordination ^#^↑ Spatial memory	^^#^↓ IL1β, TNFα, NOS2, IL12, GM-CSF ^^^↔ Oligo2+ cells ^#^↑ Oligo2+ cells
PTEN [64]	Inhibition (continuous)	M/F (pooled)	*↓ Iba1+ cells *↓ GFAP+ cells *↔ CD68+ cells	*↑ Excitatory synapses *↑ Neuronal number *↔ Synaptic connections	-	-	*↓ TNFα, IFNγ, Il1β
PTEN [65]	Inhibition (continuous)	Not stated	-	^^#^*↑ White matter integrity *↑ Cortical remapping *↑ Synaptic connections	-	^^^↓ Neurological severity score ^^#^*↑ Motor coordination ^#^*↔ Neurological severity score *↑ Spatial memory	*↑ p-Akt, p-mTOR
NLRP3 [66]	Inhibition (continuous)	M & F	*↓ Iba1 (females more than males) *↓ GFAP **(females more than males)**	*↑ Theta, gamma signalling (females more than males) *↔ Alpha, beta, delta signalling	-	^^#^*↔ Neurological severity score *↓ Depression-like behaviour	*↓ Cleaved caspase-1, Cleaved caspase-3 (females more than males) *↓ C3 (females more than males) *↑ S100A10 (females more than males) *↑ PSD95 **(females more than males)**
NLRP3 [67]	Inhibition (acute)	M	-	^#^↑ Neuronal number	^^^↑ BBB integrity ^^^↓ Oedema ^#^↓ Lesion size	*↑ Spatial memory	^^^↓ IL1β, IL18 ^^^↓ LDH
NLRP3 [68]	Inhibition (continuous)	M	-	^^^↑ Neuronal number ^#^↓ Apoptotic cells	^^^↓ Oedema ^^^↓ Lesion size	^^#^*↓ Neurological severity score ^^#^↑ Motor coordination *↔ Motor coordination	^^^↓ *Nlrp3*, *Asc*, *Caspase-1*, NLRP3, ASC, Procaspase-1, Cleaved caspase-1, Cleaved caspase-3 IL1β, IL18 ^^^↑ Occludin, Claudin-5, ZO1
GPER [69]	Activation (acute)	M/OVX (pooled)	^#^↓ Iba1+ cells ^#^↓ *Mrc1* ^#^↑ *Arg1*, *Mrc1*	-	^^^↓ Oedema ^^^↑ BBB integrity	^#^↑ Episodic memory ^#^↑ Spatial memory ^#^↓ Anxiety-like behaviour	^#^↑ *Il10*, IL10 ^#^↓ *Mcp1*, MCP1, *Nos2* ^#^↓ IL1β, CXCL2
GPER [70]	Activation (acute)	M, F, OVX	^^^↑ CD11b/CD32+ cells **(males and OVX only; females ↔)** ^^^↓ CD11b/Arg1+ cells **(males and OVX only; females ↔)**	-	-	-	^^^↓ IL1β **(males and OVX only; females ↔)** ^^^↓ IL6, IL10, TNFα **(males and OVX only; females ↑)** ^^^↑ IL4 **(males and OVX only; females ↓)** ^^^↑ GPER **(males and OVX only; females ↓)**
Adenosine A3 Receptor (ADORA3) [71]	Activation (acute)	M & F	^#^↓ *Iba1* (males and females pooled) ^#^↓ *Gfap* (males and females pooled) ^#^↓ Iba1 (males only; females not assessed) ^#^↓ GFAP (males only; females not assessed) ^#^↑ Astrocyte ATP (females only; males not assessed)	^^^↓ Cell death (males only; females not assessed)	^^^↑ BBB integrity (males only; females not assessed)	*↑ Emotional learning (males only; females ↔) *↑ Emotional memory **(males only; females had no deficit after injury)**	-
Adenosine A3 Receptor (ADORA3) [72]	Activation (acute)	M	-	-	^^^↓ Lesion size ^^^↓ Tissue disorganisation	^#^↔ Spatial memory --- *↑ Spatial memory *↔ Anxiety-like behaviour	^^^↑ IκBα ^^^↓ NFκB, p-p38, p-ERK, NLRP3, Caspase-1 ^^^↓ Peripheral myeloid infiltration
CSF1R [73]	Inhibition (acute)	M & F	^#^↓ Iba1, CD68*, Arg1, Cd68, Mrc1* ^#^↔ GFAP, *Gfap* ^#^↓ Proliferating microglia *↑ *Arg1* (females only; males ↔) *↑ *Mrc1* (females only; males ↓) *↓ CD68, *Cd68*, *Gfap* **(males only; females ↔)**	^#^↓ Apoptotic cells *↑ Neuronal number **(males only; females ↔)**	^#^↔ Lesion size ^#^↑ Haematoma size *↓ Lesion size *↓ Hippocampal volume loss **(males only; females ↔)**	^^^↓ Neurological severity score **(males only; females ↔)** ^^^*↔ Motor coordination **(males only; females** ↓) ^^#^*↔ Anxiety-like behaviour ^#^*↔ Neurological severity score	^#^↓ *Tnfa, C1qa*, *Aif*, *Fcgr1* ^#^↓ *Bax* **(males only; females ↔)** ^#^↔IL1β ^#^↔ Casp3, Il1b, Il1r1 *↔ *Il1b* **(males only; females** ↑) *↓ *Bax*, *Il1r1* **(males only; females** ↑) *↓ *Casp3* (males only; females ↔) *↔ *Aif1*, *Fcgr1*
CSF1R [74]	Delayed inhibition (continuous)	M	*↓ NOX2+/CD68+/Iba1+ cells *↓ IL1β+/Iba1+ cells *↓ Caspase-1+/Iba1+ cells *↓ “Bushy” microglia *↑ “Ramified” microglia *↔ Total Iba1+ cells *↔ GFAP	*↑ Neuronal number	*↓ Lesion size	*↑ Motor coordination *↑ Spatial memory	*↓ NLRP3 *↔ Caspase-1, P22^phox^ *↔ IL1β *↔ NCF1, NCF4 *↔ *Tnfa*, C1qa *↔ Peripheral myeloid infiltration
CSF1R [75]	Delayed inhibition (acute)	M	^#^↓ CD11b+/CD54^low^ cells ^#^↓ Iba1 *↔ Iba1	*↑ Fast-conducting neuronal connectivity *↔ Slow-conducting neuronal connectivity *↔ Dendritic complexity *↔ White matter integrity	-	*↔ Depression-like behaviour *↔ Episodic memory	-
CSF1R [76]	Inhibition (continuous)	M & F	^^^↓ CD68+ cells (males only; females not assessed) ^^^↔ CD45+/CD68- cells (males only; females not assessed) ^^^↔ GFAP (males only; females not assessed)	-	^^^↔ Lesion size ^^^↔ Haematoma size **(males only; females** ↑**)**	^^^↔ Neurological Severity Score ^^^↓ Anxiety-like behaviour	^^^↔ OPN (males only; females not assessed)
CSF1R and IL1R1 [76]	Inhibition (continuous)	M & F	^^^↓ CD68+ cells (males only; females not assessed) ^^^↓ CD45+/CD68- cells (males only; females not assessed) ^^^↔ GFAP (males only; females not assessed)	-	^^^↓ Lesion size **(males only; females ↔)** ^^^↔ Haematoma size	^^^↓ Neurological Severity Score ^^^↓ Anxiety-like behaviour	^^^↓ OPN (males only; females not assessed)
IL1R1 [76]	Inhibition (continuous)	M & F	^^^↓ CD68+ cells (males only; females not assessed) ^^^↔ CD45+/CD68- cells (males only; females not assessed) ^^^↔ GFAP (males only; females not assessed)	-	^^^↔ Lesion size ^^^↑ Haematoma size	^^^↓ Neurological Severity Score **(females only, males ↔)** ^^^↓ Anxiety-like behaviour	^^^↔ OPN (males only; females not assessed)
IL1R1 [77]	Inhibition (continuous)	M	*↓ Microglia density *↓ Astrocyte density	-	-	*↑ Emotional memory	-
IL1R1 [78]	Inhibition (continuous)	M/F (pooled)	^^^↓ Microglia density ^^^↓ Astrocyte density --- ^#^↔ Microglia density ^#^↔ Astrocyte density	^^#^↓ Axonal damage	^^^↔ Lesion size	^#^*↔ Emotional memory	^^^↓ Peripheral myeloid infiltration ^^^↑ *Cd68*, *Irf7*, *Tnf* ^^^↔ *Il1b*, *H2eb1* --- ^#^↔ *Cd68*, *Irf7*, *Il1b* ^#^↓ *Tnf*, *H2eb1* --- *↓ *Tnf*, Tlr2 *↔ *Cd68*, *Irf7*, *C1qa*
PPARγ [79]	Activation (continuous)	M/F (pooled)	*↓ Iba1, CD68 *↓ GFAP	-	-	*↑ Spatial memory	*↑ PGC1α *↓ p-p65, p-STAT3, IL6, NLRP3, TNFα
PPARγ [80]	Activation (acute)	M	^^^↓ GFAP ^^^↓ C3+/GFAP+ cells	^^^↓ Degenerating neurons	^^^↓ Oedema	^#^↑ Motor coordination	^^^↑ TGFβ ^^^↓ TNFα, IL1, IL10 ^^^↓ C3, C1q

Outcomes are summarised in rows alongside an arrow and timepoint marker. Arrows indicate that outcome in experimental TBI model was significantly increased (↑), decreased (↓) or unchanged (↔) compared to control TBI of the same sex. Timepoint markers indicate outcome across days post-injury (DPI): ^^^ = Acute (≤3 DPI); ^#^ = Subacute (4–14 DPI); * = Chronic (≥15 DPI). Therapies applied for <1 week were categorised as an acute dose; long-term dosing (≥1 week) or genetic knockout models were categorised as continuous. Abbreviations: M = male; F = female; OVX = ovariectomised female.

**Table 2:** Summary of clinical trials evaluating therapies targeting neuroinflammation after traumatic brain injury in which biological sex was inadequately considered during study design, analysis, or interpretation.

Clinical Trial	Study Design	Female Participants in Treatment Arm	Intervention	Primary Endpoint(s)	Main Outcome	Sex-Stratified Analysis?
EPO-TBI (NCT00987454) [111]	Multinational, randomised, double-blind, placebo-controlled Phase III trial	49/305 (16.1%)	Subcutaneous injection of Erythropoietin-α within 24 h of injury, administered weekly for up to 3 doses.	GOSE at 6 months	No benefit	No
SYNAPSE (NCT01143064) [112]	Multinational, randomised, double-blind, placebo-controlled Phase III trial	127/591 (21.5%)	Intravenous progesterone within 8 h of injury given for a total of 120 h	GOS at 6 months	No benefit	No
ProTECT III (NCT00822900) [113]	Multicentre, randomised, double-blind, placebo-controlled Phase III trial	118/442 (26.7%)	Intravenous progesterone within 4 h of injury given for a total of 96 h	GOSE at 6 months	No benefit	Yes
Tirilazad Mesylate Phase III Trial [114]	Multinational, randomised, double-blind, placebo-controlled Phase III trial	137/562 (24.4%)	Intravenous tirilazad mesylate within 4 h of injury, administered every 6 h for 5 days	GOS at 6 months	No benefit	Yes
Doxycycline in Acute Traumatic Brain Injury (NCT04239755) [115]	Randomised, double-blind, placebo-controlled Phase II trial	12/25 (48%)	Oral or enteral administration of doxycycline within 24 h of injury, administered twice daily for 7 days	NSE serum levels at 7 days	Significantly decreased NSE serum levels	No
Minocycline effects in patients with traumatic brain injury (IRCT201602063014N12) [116]	Randomised, double-blind, placebo-controlled Phase II trial	2/14 (14.3%)	Oral administration of minocycline after surgical evacuation of haematoma, administered every 12 h for 7 days	S100B serum levels at 1–4 days; NSE serum levels at 1, 3 and 5 days	No S100B difference; significantly reduced NSE levels between days 1–5	No
NOSTRA-III (NCT02794168) [117]	Multinational, randomised, double-blind, placebo-controlled Phase III trial	Not yet reported	Intravenous Ronopterin administered for 48 h	GOSE at 6 months	No benefit	Not yet reported

Abbreviations: GOSE = Glasgow Outcome Scale-Extended, GOS = Glasgow Outcome Scale, NSE = Neuron-Specific Enolase.

## Data Availability

No new data were created or analyzed in this study. Data sharing is not applicable to this article.

## Abbreviations

The following abbreviations are used in this manuscript:

TBI	Traumatic brain injury
NMDA	N-methyl-D-aspartate receptor
AMPA	α-amino-3-hydroxy-5-methyl-4-isoxazolepropionic acid
ATP	Adenosine triphosphate
ROS	Reactive oxygen species
BBB	Blood-brain barrier
DAMPs	Damage-associated molecular patterns
CNS	Central nervous system
IRFs	Interferon regulatory factors
STATs	Signal transducers and activators of transcription
NF-κB	Nuclear factor kappa-light-chain-enhancer
TNF-α	Tumour necrosis factor α
IL	Interleukin
IGF-1	Insulin-like growth factor 1
VEGF	Vascular endothelial growth factor
ADORA3	Adenosine A3 Receptor
DPI	Days post injury
OVX	Ovariectomised
MEK	Mitogen-activated protein kinase kinase
ERK	Extracellular signal-regulated kinase
MAPK	p38 mitogen-activated protein kinase
MKK	MAP kinase kinase
GPER	G protein-coupled oestrogen receptor
cAMP	Cyclic adenosine monophosphate
PKA	Protein kinase A
CREB	Cyclic AMP response element-binding protein
PI3K	Phosphoinositide 3-kinase
Akt	Protein kinase B
CSF1R	Colony-stimulating factor 1 receptor
NLRP3	Nucleotide-binding oligomerisation domain-like receptor protein 3
GOSE	Glasgow outcome scale-extended
GOS	Glasgow outcome scale
NSE	Neuron-specific enolase
ANZICS-CTG	Australian and New Zealand Intensive Care Society Clinical Trials Group
EPO	Erythropoietin
